# 55 Risk of Sepsis and Mortality in SJS and TENS Patients Treated with Corticosteroids

**DOI:** 10.1093/jbcr/irad045.029

**Published:** 2023-05-15

**Authors:** Catherine Everett, Ashley Hink, Steven Kahn, Deepak Ozhathil

**Affiliations:** Medical University of South Carolina, Charleston, South Carolina; Medical University of South Carolina, Charleston, South Carolina; The South Carolina Burn Center/Medical University of South Carolina, Charleston, South Carolina; South Carolina Burn Center at MUSC, Charleston, South Carolina; Medical University of South Carolina, Charleston, South Carolina; Medical University of South Carolina, Charleston, South Carolina; The South Carolina Burn Center/Medical University of South Carolina, Charleston, South Carolina; South Carolina Burn Center at MUSC, Charleston, South Carolina; Medical University of South Carolina, Charleston, South Carolina; Medical University of South Carolina, Charleston, South Carolina; The South Carolina Burn Center/Medical University of South Carolina, Charleston, South Carolina; South Carolina Burn Center at MUSC, Charleston, South Carolina; Medical University of South Carolina, Charleston, South Carolina; Medical University of South Carolina, Charleston, South Carolina; The South Carolina Burn Center/Medical University of South Carolina, Charleston, South Carolina; South Carolina Burn Center at MUSC, Charleston, South Carolina

## Abstract

**Introduction:**

Stevens-Johnson Syndrome (SJS) and Toxic Epidermal Necrolysis Syndrome (TENS) are a rare adverse cutaneous drug reaction that historically has a high risk of mortality. The use of corticosteroids (CS) to treat SJS/TENS remains controversial as the literature is limited to small cohort trials by the rarity of the condition. Some conflicting studies suggested that CS may increase mortality due to augmented infection risk. This study evaluated the incidence of sepsis and mortality following CS use in patients with SJS/TENS.

**Methods:**

We performed a retrospective study utilizing a commercially available multi-institutional database derived from deidentified inpatient coding data to assess the incidence of sepsis and mortality within 30 days after diagnosis of SJS/TENS in patients >18 years old treated with CS within one week of their initial diagnosis. Data was analyzed from years 2000-2022. Patients treated with CS within 1 week of diagnosis (n=3930) and those treated without CS (n=6557) were propensity matched for age, race, gender, severe sepsis and SIRS (n=3723) and were assessed for relative risk of mortality and sepsis occurrence.

**Results:**

Of the patients who received CS 171 of 3723 died vs 98 of 3723 of those that did not receive CS. They incurred a 1.961% relative risk (RR: 1.745, 1.367, 2.227 95% CI, p=< 0.0001) for mortality. Similarly, of the patients that received CS 245 of 3723 suffered sepsis vs 173 of 3723 that did not receive CS. This was a 1.934% relative risk (RR: 1.416, 1.172, 1.711 95% CI, p=0.0003) of developing sepsis. The number of instances and median days till instance were not statistically different between cohorts.

**Conclusions:**

This study indicated that amongst adult patients diagnosed with SJS/TENS, the utilization of CS within the first week of diagnosis is affiliated with a statistically significant increased risk of sepsis and death within 30 days of diagnosis.

**Applicability of Research to Practice:**

The application of CS therapy may enable the development of infections in patients such as sepsis, which may ultimately worsen prognosis, but the rarity of this condition prohibits effective single-institution assessment of this impact. Care should be taken to assess the risks and benefits of prescribing CS therapy in SJS/TENS patients.

